# A Discrete Choice Analysis Comparing COVID-19 Vaccination Decisions for Children and Adults

**DOI:** 10.1001/jamanetworkopen.2022.53582

**Published:** 2023-01-30

**Authors:** Lisa A. Prosser, Abram L. Wagner, Eve Wittenberg, Brian J. Zikmund-Fisher, Angela M. Rose, Jamison Pike

**Affiliations:** 1 University of Michigan, Ann Arbor; 2 Harvard Chan School of Public Health, Boston, Massachusetts; 3 Centers for Disease Control and Prevention, Atlanta, Georgia

## Abstract

**Importance:**

COVID-19 vaccination rates in the US remain below optimal levels. Patient preferences for different attributes of vaccine products and the vaccination experience can be important in determining vaccine uptake decisions.

**Objective:**

To assess preferences for attributes of adult and pediatric COVID-19 vaccination among US adults.

**Design, Setting, and Participants:**

An online survey of a national panel of 1040 US adults was conducted in May and June 2021. A discrete choice analysis was used to measure the relative value of each attribute in the decision to choose a COVID-19 vaccination option for adults or children. Six attributes were used to described hypothetical vaccination options: vaccine effectiveness, mild side effects, rare adverse events, number of doses, time required for vaccination, and regulatory approval. Respondents chose between hypothetical vaccination profiles or no vaccination. Additional survey questions asked about vaccination beliefs, COVID-19 illness experience, COVID-19 risk factors, vaccination status, and opinions about the risk of COVID-19.

**Exposures:**

Respondents chose which vaccine profile they would prefer to receive for themselves (or no vaccination). Respondents then considered an identical set of profiles for a hypothetical child aged 0 to 17 years.

**Main Outcomes and Measures:**

Relative value of vaccination-related attributes were estimated using Bayesian logit regression. Preference profiles for subgroups were estimated using latent class analyses.

**Results:**

A total of 1040 adults (610 [59%] female; 379 participants [36%] with an age of 55 years and older years) responded to the survey. When asked about vaccination choices for themselves, participants indicated that vaccine effectiveness (95% vs 60%) was a significant attribute (β, 9.59 [95% CrI, 9.20-10.00] vs β, 0.41 [95% CrI, 0-0.80]). Respondents also preferred fewer rare adverse events (β, 6.35 [95% CrI, 5.74-6.86), fewer mild side effects (β, 5.49; 95% CrI, 5.12-5.87), 1 dose (β, 5.41; 95% CrI, 5.04-5.78), FDA approval (β, 6.01; 95% CrI, 5.64-6.41), and shorter waiting times (β, 5.67; 95% CrI, 4.87-6.48). Results were very similar when framing the question as adult or child vaccination, with slightly stronger preference for fewer rare adverse events for children. Latent class analysis revealed 4 groups of respondents: (1) individuals sensitive to safety and regulatory status, (2) individuals sensitive to convenience, (3) individuals who carefully considered all attributes in making their choices, and (4) individuals who rejected the vaccine.

**Conclusions and Relevance:**

In this survey study of US adults, the identification of 4 distinct preference groups provides new information to guide communications to support vaccine decision making. In particular, the group that prioritize convenience (less time required for vaccination and fewer doses) may present an opportunity to create actionable strategies to increase vaccination uptake for both adult and pediatric populations.

## Key Points

**Question:**

Do preferences for COVID-19 vaccination vary across pediatric and adult vaccination?

**Findings:**

In this survey study of 1040 US adults, all included attributes (vaccine effectiveness, mild side effects, rare adverse events, number of doses, regulatory approval, waiting time) were important factors in choosing to vaccinate with little difference in magnitude when comparing adult and pediatric vaccination. Latent class analyses identified 4 distinct subgroups, including 1 subgroup sensitive to convenience (waiting time and number of doses).

**Meaning:**

These findings suggest that providing more convenient vaccination options may represent an actionable strategy to increase vaccination uptake for a subgroup of the population.

## Teaser

This survey study assesses preferences for attributes of adult and pediatric COVID-19 vaccination among US adults.

## Introduction

During 2020 to 2021, 3 vaccine products were authorized for emergency use for the prevention of severe illness from COVID-19.^1,2,3^ Initial emergency use authorization was for adults, with extended authorization to children aged 12 to 17 for 1 mRNA vaccine in May 2021.^4^ Vaccination coverage for adolescents lags behind that for adults.^5^ Additional authorizations for children age 5 to 11 years and 6 months to 4 years were approved in November 2021 and June 2022, respectively.^6^ Understanding vaccination decision making for the pediatric age group will be important as vaccination continues and expands to younger ages.

This study used conjoint analysis, a survey-based approach for measuring preferences.^7^ The premise of conjoint analysis is that any good or service can be described by a set of attributes and when using choice-based survey questions it is possible to quantify and measure the relative value of each attribute in the decision to choose a good or service. Conjoint analysis was originally developed for marketing and transportation analysis and is increasingly being used to understand individual decision making for health interventions and public health programs.^8,9,10,11^ This study used a specific type of conjoint analysis, discrete choice analysis, in which survey respondents are asked to choose between profiles of attributes that describe a vaccination option.

Surveys using a conjoint analysis and discrete choice experiment (DCE) approach have measured the importance of COVID-19 vaccine effectiveness and safety,^12,13^ vaccination attributes, such as fewer doses and shorter waiting times,^12,14^ in vaccination preferences for US adults. These studies did not assess preferences for pediatric vaccination. This survey used a discrete choice experiment approach to quantify the relative value of vaccination attributes on vaccine uptake decisions for both adults and children

The objective of this study was to measure any differences in preferences for vaccination among pediatric and adult populations. Previous studies of vaccine-preventable outcomes have reported higher values for averting illness in children compared with adults.^15,16^ Other vaccination preference studies have documented increasing rates of vaccine hesitancy for parents with the risk of adverse events as 1 of many concerns.^17^ Higher values for averting illness in children would predict higher rates of vaccination for children, but given the emergency use authorization approval status and extensive literature on increasing visibility of vaccine hesitancy for routine childhood vaccination and influenza among parents pre-COVID-19,^17,18,19,20^ our hypothesis was that preferences would differ between adult and child vaccination for mild side effects, rare adverse events, and number of doses.

## Methods

This survey study used a discrete choice experiment to measure the relative value of vaccine- and vaccination-attributes in decision making for COVID-19 vaccines. This study was reviewed and determined to be exempt from ongoing review by the institutional review board of the University of Michigan Medical School. Survey data were deidentified and a waiver of informed consent documentation was also granted by the institutional review board. The survey follows the American Association for Public Opinion Research (AAPOR) reporting guideline, and this article reports the disclosure elements described in the AAPOR’s Transparency Initiative.^21,22^

### Survey Design and Development

**Figure 1.**
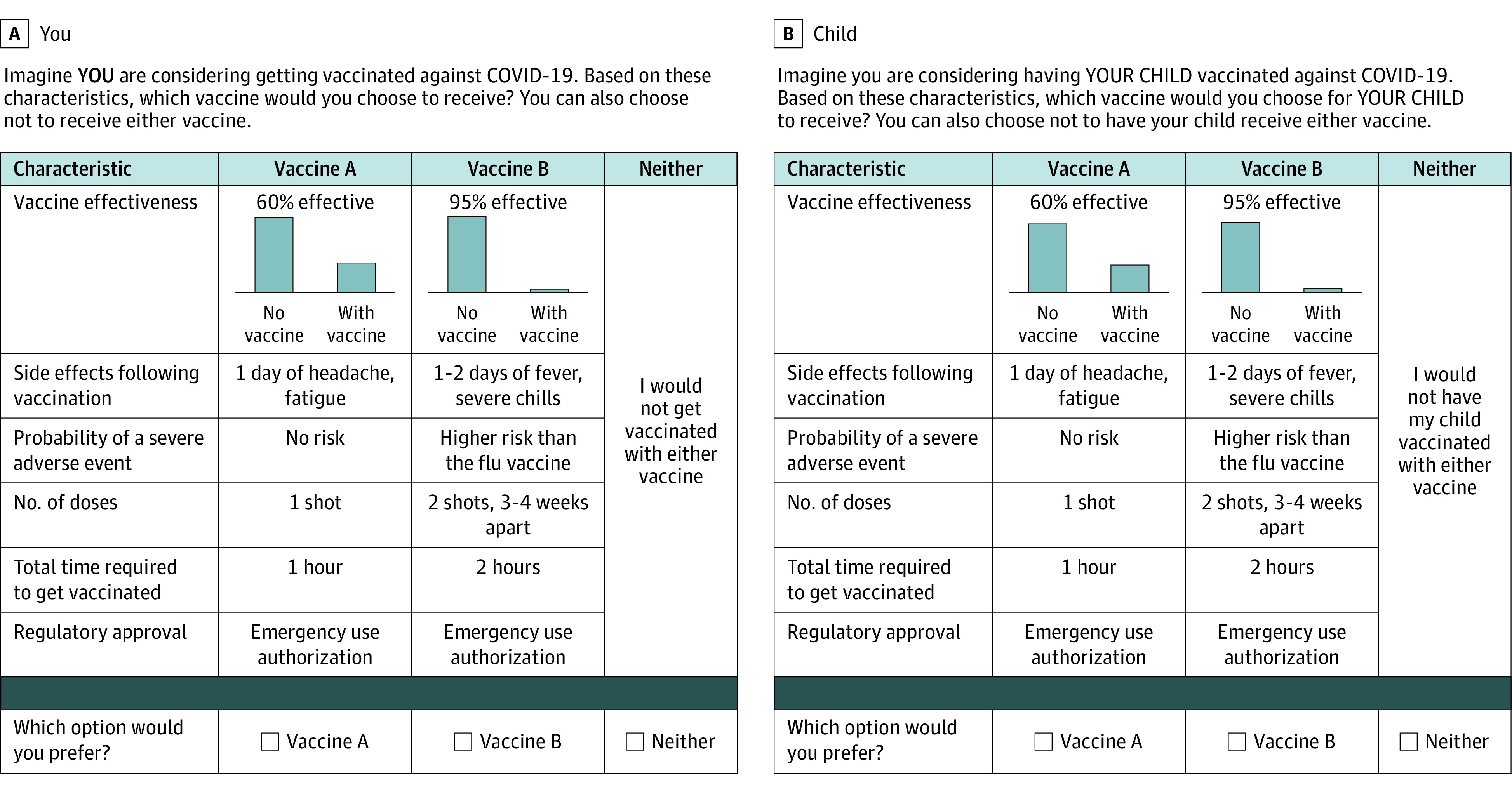
Example Choice Questions

The survey instrument included an introductory section, 2 practice choice questions, 6 choice questions (3 adult, 3 child), sociodemographic questions (including gender, age, race and ethnicity, education, and household income), and attitudes toward vaccination and COVID-19 illness. Race and ethnicity were self-identified and included because vaccine decisions were shown to be associated with race and ethnicity during the COVID-19 pandemic. Choice questions included 2 vaccination profiles and an opt-out option. Each vaccination profile contained the following attributes: (1) vaccine effectiveness of 60% or 95%; (2) mild common side effects (1 day mild or 1 to 2 days systemic); (3) rare but severe side effects (none, same risk as influenza vaccine, higher risk than influenza vaccine); (4) number of doses (1 dose or 2 doses); (5) total time required to get vaccinated; and (6) regulatory approval (emergency use authorization or full regulatory approval) (Table 1). Attributes of vaccination were chosen based on existing literature of important factors for vaccination decisions and focus of public discussion of COVID-19 vaccination.^12,23^ Descriptions and levels were developed using information available for vaccine products in development in winter 2020 to 2021, spring 2021, and prior to the availability of clinical trial data on pediatric vaccination. An example of a choice question is shown in Figure 1. Hypothetical vaccination profiles were generated using a fractional factorial design^24^ in SAS version 9.4 (SAS Institute).

**Table 1.** Discrete Choice Experiment Attributes, Descriptions, and Levels as Described in the Survey Instrument

Characteristic	Description	Levels
Vaccine effectiveness	Vaccine effectiveness is how much the vaccine reduces the risk of COVID-19 illness.	60% 95%
Mild common side effects	Each vaccine may have different levels of mild side effects.	1 day of headache, fatigue 1-2 days of fever, severe chills
Rare but severe side effects	Vaccination may be associated with rare but severe side effects. These severe side effects could include a severe allergic reaction or a serious permanent disability that affects your nervous system following vaccination.	No risk Same risk as the flu vaccine Higher risk than the flu vaccine
No. of doses	The number of doses is the number of shots you need to receive to be protected.	1 shot 2 shots, 3-4 weeks apart
Total time required to get vaccinated per dose	The total time required to get vaccinated including travel, waiting, and vaccination time for 1 dose of the vaccine.	1 hour 2 hours 4 hours 8 hours
Regulatory approval	Regulatory approval is FDA approval of the vaccine. Full review and FDA approval typically occurs after the completion of long-term research studies (Phase 3 clinical trials) over several years. The FDA may also grant emergency use authorization where the vaccine will be made available to the public prior to the completion of clinical trials and prior to full review by FDA.	Formal FDA approval following completion of the clinical trial Emergency use authorization

Abbreviation: FDA, Food and Drug Administration.

Respondents answered 2 practice discrete choice questions that were not scored and then a total of 6 scored discrete choice questions. Consistent with our primary objective to compare vaccination preferences in adults vs children, the first set of 3 discrete choice questions asked about vaccination preferences for the respondents themselves and the next 3 discrete choice questions presented the exact same set of profiles but framed as vaccination for their actual or hypothetical child under the age of 18 years. Attitudinal questions included respondent’s beliefs about vaccination in general, previous COVID-19 illness, COVID-19 risk factors, COVID-19 vaccination status,^25^ and opinions about the risks associated with COVID-19. Respondents were asked to rate their level of confidence in their answers to the DCE questions. Finally, respondents indicated if they were thinking of a child of a certain age when answering the DCE questions. All responses to questions, including for demographic questions like race and ethnicity were based on participant self-report. Survey development included pretesting with individual key informant interviews. Using a standardized pretesting discussion guide, 6 iterative versions of the survey were pretested among 11 respondents.

### Study Population and Data Collection

The survey was administered to a standing panel of US adults from May 21, 2021, to June 9, 2021, from the Qualtrics^26^ Online Panel (n = 1040). At the time of the survey, emergency use authorization had been granted for adolescents aged 12 to 15 years, but younger children were not approved for vaccination.^27^ Vaccinations with both 1- and 2-dose series were available, primarily in a mass vaccination setting often with long wait times. The median completion time for the survey was 7.7 minutes. Respondents could skip questions. Any respondents who ended the survey early were excluded from the analysis. Respondents who completed the survey received an incentive from the survey vendor in the form of points that can be used toward gift cards and other rewards.

### Statistical Analyses

Analysis was conducted using SAS version 9.4 (SAS Institute), along with the LCA extension (The Methodology Center).^28^ The primary analysis used a Bayesian logit model,^29^ with attribute levels entered as categorical variables with effects coding.^30^ This analysis used noninformative priors and output posteriors with a 95% credibility interval (CrI) based on the highest posterior density.^28,30^ We conducted all analyses separately, first for participants considering vaccination as an adult and then for participants considering childhood vaccination. The primary analysis included the full sample. A secondary analysis excluded those who reported low confidence in their responses to the choice questions (n = 8). Additional analyses evaluated differences in preferences based on COVID-19 attitudes, vaccination behaviors, and experiences.

We conducted a latent class analysis to identify unobserved subgroups with similar preferences, testing a range of class sizes (3-5) to determine the best model fit. The final set of latent classes selected was based on a consideration of model fit but also interpretability of results. We assessed the significance of class membership and demographics and COVID-related factors using χ^2^ tests, with significance set at an α level of .05. Additional analyses compared the odds of opting out of the profiles (ie, rejecting vaccine) by sociodemographic group. We compared decision making for opting out between the adult and childhood profiles by merging the adult and childhood data sets together and specifying an interaction term between demographic characteristics and a dummy variable for the data set type. Incomplete responses (n = 82) were not included in the analysis. Demographic data for these respondents were not provided.

## Results

**Figure 2.**
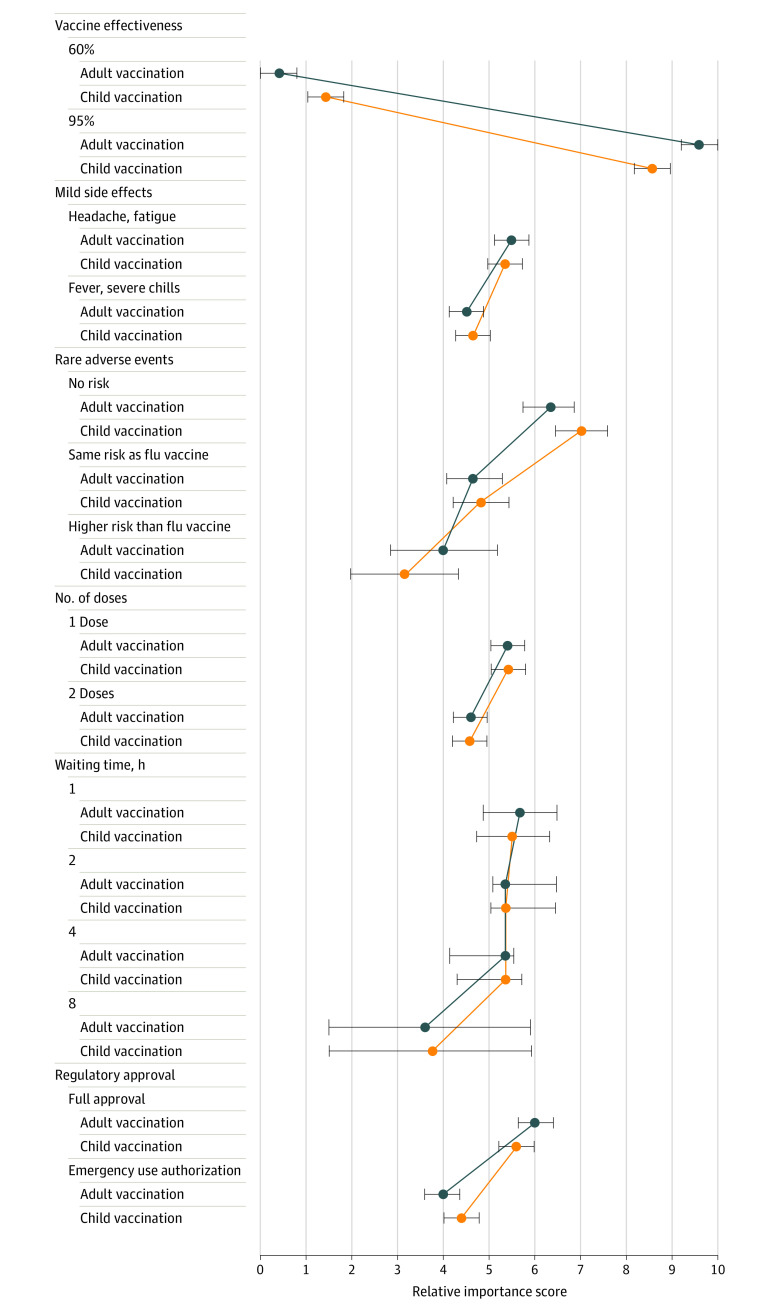
Relative Preferences for Vaccination Attributes and Levels, Comparing Adult and Child Vaccination

Respondents of this survey study of 1040 adults reflected characteristics of the adult US population except with 610 (59%) of participants being female, which is higher than 51% for the US adult population at large,^31^ and 379 (36%) with an age of 55 years and older (eTable 1 in Supplement 1). In the primary analysis, all attributes were significant for both adult and child vaccination for those who selected vaccination. Vaccine effectiveness (95% vs 60%) was the most important attribute with a significant preference for higher vaccine effectiveness. All other attributes were significant but had less influence on respondents’ choices. Respondents also preferred fewer rare adverse events, fewer common side effects, 1 dose, FDA approval, and less waiting time (Figure 2 and Table 2).

**Table 2.** Relative Preferences for Vaccination Attributes and Levels, Comparing Adult and Child Vaccination, Regression Results

Characteristic	β (95% CrI)	
	Adult	Child
Vaccine effectiveness		
60%	0.41 (0-0.80)	1.44 (1.04-1.82)
95%	9.59 (9.20-10.0))	8.56 (8.18-8.96)
Mild side effects		
Headache	5.49 (5.12-5.87)	5.35 (4.97-5.73)
Fever, severe chills	4.51 (4.13-4.88)	4.65 (4.27-5.03)
Rare adverse events		
No risk	6.35 (5.74-6.86)	7.01 (6.45-7.59)
Same risk as flu vaccine	4.65 (4.08-5.29)	4.80 (4.22-5.44)
Higher risk than flu vaccine	4.00 (2.85-5.18)	3.19 (1.97-4.33)
Number of doses		
1 dose	5.41 (5.04-5.78)	5.42 (5.05-5.80)
2 doses	4.59 (4.22-4.96)	4.58 (4.20-4.95)
Regulatory approval		
Full approval	6.01 (5.64-6.41)	5.61 (5.22-5.99)
Emergency use authorization	3.99 (3.59-4.36)	4.39 (4.01-4.78)
Waiting time		
1 hour	5.67 (4.87-6.48)	5.51 (4.73-6.33)
2 hours	5.36 (5.08-6.47)	5.37 (5.04-6.45)
4 hours	5.36 (4.14-5.54)	5.36 (4.30-5.72)
8 hours	3.60 (1.50-5.91)	3.76 (1.51-5.93)

Results were very similar when framing the question as adult or child vaccination, with slightly stronger preference for fewer rare adverse events in children. For number of doses and waiting time, responses were almost identical for adults and children. For rare adverse events, there was a trend for higher preference for no risk for children compared to adults. Vaccine effectiveness of 95% vs 60% was strongly preferred for both adults and children, with slightly stronger preference in adults (Figure 2; and eTable 1 in Supplement 1).

Subgroup analyses by COVID-19 attitudes or behaviors showed similar results. Those who did not intend to vaccinate place a higher weight on no risk of adverse events for both children and adults compared with those who intend to vaccinate (eTables 3-6 and eFigures 2-4 in Supplement 1). Secondary analyses which excluded respondents with low confidence in their responses also showed similar results (eTable 2 and eFigure 1 in Supplement 1).

**Figure 3.**
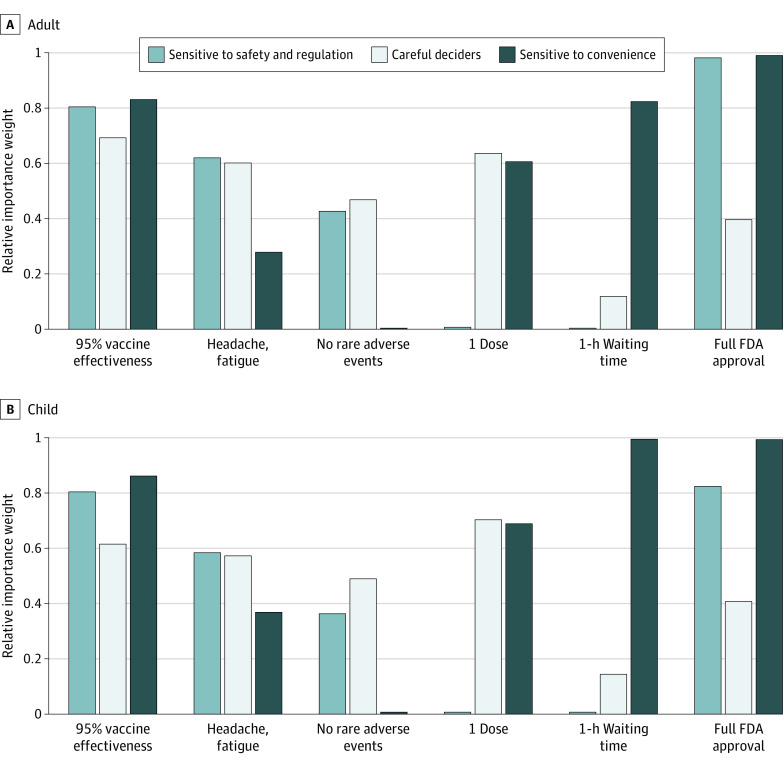
Relative Importance Weights for Choice Attributes (Latent Class Analysis)

Latent class analysis revealed 4 groups of respondents: (1) sensitive to safety and regulatory status (adverse events, FDA approval), (2) sensitive to convenience (number of doses, waiting time), (3) respondents who considered all attributes in making their choices, and (4) respondents who opted out of taking the vaccine (Figure 3; eFigure 5 in Supplement 1). These latent classes were identified for both adult and child vaccination frames. Respondents who considered all attributes in making their choices were the largest group for both, with 57% in this category for adult vaccination and 48% for child vaccination. Those focused on safety and regulation were slightly more represented in the child vaccination frame at 18% compared with 14% in adults. There were also smaller but clearly defined group of convenience-focused respondents for both adult vaccination (9%) and child vaccination (6%) (eTables 6-8 in Supplement 1). Respondents chose to opt-out of 21% of adult vaccination choices and 29% of child vaccination choices. In general, the decision to opt-out to adult and childhood vaccines had similar patterns across demographic groups (eTable 9 in Supplement 1). An exception was that respondents who were thinking of young children (ie, aged 0-5 years) were more likely to opt out of a vaccine for a child compared to a vaccine for themselves.

## Discussion

This survey study reports results from a US adult sample directly comparing vaccination preferences for adult and child vaccination against COVID-19 using a survey approach, discrete choice experiment, designed to quantitatively measure relative values for choice attributes. Among 6 vaccine attributes, vaccine effectiveness was a significant attribute, followed by the risk of rare severe adverse events. Results were similar when comparing choices across adult and child vaccination, with slightly stronger preference for fewer severe adverse events and full regulatory approval in children compared with adults. People who prioritize convenience (ie, less time required for vaccination and fewer doses) may represent an opportunity for actionable strategies to increase vaccination uptake for both adult and pediatric populations.

Consistent with the extensive literature on vaccine decision making, there is no single or primary reason for vaccine hesitancy. Vaccine acceptance represents a spectrum ranging from decliners of all vaccines to unquestioning acceptors.^17^ Similarly, the reasons for declining COVID-19 vaccination in the moment represents a vast range of beliefs and attitudes from decliners to acceptors. In between, there will be individuals who desire more information, more assistance with decision making, and various combinations of these strategies for support. In this study, the largest group identified in the latent class analysis consisted of respondents who considered all attributes in making their choices, suggesting that strategies that help individuals deliberate could be beneficial. The smallest group, sensitive to convenience, may represent the most straightforward group to address. For this subgroup, strategies that bring vaccination directly to neighborhoods or even door-to-door may be effective in increasing vaccine uptake. A recent survey in adults identified a latent class concerned about service delivery representing 8.8% of the sample.^14^ Our study confirms this subgroup for adults and further identifies a similar subgroup with focus on convenience for child vaccination. Future studies could assess if this subgroup exists for other vaccinations. Other studies including latent class analyses have shown similar classifications of individuals. For example, a study from Australia found 1 class of individuals predominantly concerned with timing and place of vaccination (similar to our convenience-focused class), 1 broadly considering all options, and another with relatively more concerns about safety than other attributes.^32^

Existing discrete choice experiments and other stated preference studies evaluating vaccination choices have shown similar preference profiles with a strong emphasis on vaccine effectiveness but focused only on adult vaccination. In this study, vaccine effectiveness was also identified as a significant attribute. Similarly, a cross-national study from the US and several Asian countries found 75% to 76% acceptance of a 50% effective vaccine, vs 88% to 89% acceptance of a 95% effective vaccine.^33^ A study conducted in the US before the roll-out of the vaccines also found that reductions in vaccine effectiveness contributed the most to profile choice.^34^

This study contributes to the literature by adding the comparison to child vaccination. Respondents were more likely to opt out of vaccination when considering child vaccination (29% of profiles) compared with adult vaccination (21% of profiles). This is consistent with cross-sectional surveys that report lower intentions for vaccination in children,^35^ especially younger children. Reduced intention to vaccinate children has increased over time. For example, the Kaiser Family Foundation survey found 22% of parents would not vaccinate a 12 to 17-year old child in April 2021, compared to 31% in October 2021; between July and October 2021, those who would definitely not vaccinate a 5 to 11-year old child increased from 25% to 30%.^36^ As of December 2021, 60% of adolescents age 12 to 15 years and 66% aged 16 to 17 had received at least 1 dose of a COVID-19 vaccine compared with 16.7% of children aged 5 to 11 years old.^37,38^

Importantly, decisions for adolescent and childhood vaccination must be considered in the context of joint decision making between child and parent or caregiver. Especially for adolescents, there is likely to be a continuum of possible contexts for the inclusion of both child and parent or caregiver preferences.^39,40^ There have been some cases reported in the media of adolescents secretly obtaining access to vaccinations when parents are withholding consent.^41^

### Limitations

This survey study has limitations. The survey was conducted in the late spring and early summer of 2021 when circulating virus differed from the levels currently observed. Only a limited set of vaccination attributes was included to keep the survey to a feasible set for respondents. Additional factors have also been shown to be influential in decisions for vaccine other than COVID-19. For example, previous surveys suggest clinician recommendation is a highly ranked attribute for vaccination decisions for influenza and this could be an important factor for many individuals and parents.^23^ At the time of this survey, most vaccinations for COVID-19 were occurring outside of the clinician office setting, but as the need for additional booster doses is considered and vaccination moves into the clinician offices, the role of clinician recommendation will gain importance. The study included some respondents who do not have their own children. The order of age groups or attributes was not randomized in the survey presentation. The size of the convenience subgroup is relatively small (6%-9% of the overall sample). Future studies should explore the characteristics of this group including predicted uptake for convenience-based vaccination strategies. Finally, this survey was conducted online, excluding those who do not have access to the internet who may have had different preferences for COVID-19 vaccination.

## Conclusions

The findings of this survey study suggest that the identification of a subgroup who prioritizes convenience (lower time required for vaccination and fewer doses) may present an opportunity for strategies to increase vaccination uptake both for vaccination against COVID-19 but also for other vaccines. The highly increased availability of vaccination at mass vaccination sites and pharmacies provides important opportunities for increasing vaccine uptake. Strategies that provide even more convenient vaccination access, such as school-located clinics, drive-through clinics, neighborhood pop-ups, or even door-to-door vaccination, represent potential strategies to improve vaccination coverage.

## Notes

Supplement 1. **eTable 1.** Respondent Characteristics **eTable 2.** Subgroup Analysis **eTable 3.** Relative Preferences for Vaccination Attributes and Levels, Regression Results, Comparing Intent to Vaccinate **eTable 4.** Relative Preferences for Vaccination Attributes and Levels, Comparing COVID-19 Beliefs—Regression Results **eTable 5.** Relative Preferences for Vaccination Attributes and Levels, Comparing COVID-19 Experience—Regression Results **eTable 6.** Latent Class Analysis Showing the Proportion Belonging to Each Class, Along With the Proportion Within Each Class Choosing a Profile Based on the Presence of a Specific Attribute Level **eTable 7.** Relative Importance Weights for Choice Attributes (Latent Class Analysis) **eTable 8.** Latent Class Analysis by Respondent Characteristics **eTable 9.** The Odds of Opting Out by Demographic and COVID-19-Related Experiences, Stratified by Decision-Making for Oneself vs for a Child **eFigure 1.** Relative Preferences for Vaccination Attributes and Levels in Limited Population, Comparing Adult and Child Vaccination **eFigure 2.** Relative Preferences for Vaccination Attributes and Levels, Comparing Intent to Vaccinate **eFigure 3.** Relative Preferences for Vaccination Attributes and Levels, Comparing COVID-19 Beliefs **eFigure 4.** Relative Preferences for Vaccination Attributes and Levels, Comparing COVID-19 Experience **eFigure 5.** Latent Class Analysis

Supplement 2.
Data Sharing Statement
