# Supplementary material for: A Discrete Choice Analysis Comparing COVID-19 Vaccination Decisions for Children and Adults
Source: JAMA Netw Open. 2023 Jan 30;6(1):e2253582. doi: 10.1001/jamanetworkopen.2022.53582 (PMC9887501; doi:10.1001/jamanetworkopen.2022.53582)
Supplement: Supplement 1. — eTable 1. Respondent Characteristics eTable 2. Subgroup Analysis eTable 3. Relative Preferences for Vaccination Attributes and Levels, Regression Results, Comparing Intent to Vaccinate eTable 4. Relative Preferences for Vaccination Attributes and Levels, Comparing COVID-19 Beliefs—Regression Results eTable 5. Relative Preferences for Vaccination Attributes and Levels, Comparing COVID-19 Experience—Regression Results eTable 6. Latent Class Analysis Showing the Proportion Belonging to Each Class, Along With the Proportion Within Each Class Choosing a Profile Based on the Presence of a Specific Attribute Level eTable 7. Relative Importance Weights for Choice Attributes (Latent Class Analysis) eTable 8. Latent Class Analysis by Respondent Characteristics eTable 9. The Odds of Opting Out by Demographic and COVID-19-Related Experiences, Stratified by Decision-Making for Oneself vs for a Child eFigure 1. Relative Preferences for Vaccination Attributes and Levels in Limited Population, Comparing Adult and Child Vaccination eFigure 2. Relative Preferences for Vaccination Attributes and Levels, Comparing Intent to Vaccinate eFigure 3. Relative Preferences for Vaccination Attributes and Levels, Comparing COVID-19 Beliefs eFigure 4. Relative Preferences for Vaccination Attributes and Levels, Comparing COVID-19 Experience eFigure 5. Latent Class Analysis [file jamanetwopen-e2253582-s001.pdf]

Supplemental Online Content

Prosser LA, Wagner AL, Wittenberg E, Zikmund-Fisher BJ, Rose AM, Pike J. A discrete choice analysis comparing COVID-19 vaccination decisions for children and adults. *JAMA Netw Open*. 2023;6(1):e2253582. doi:10.1001/jamanetworkopen.2022.53582

eTable 1. Respondent Characteristics

eTable 2. Subgroup Analysis

eTable 3. Relative Preferences for Vaccination Attributes and Levels, Regression Results, Comparing Intent to Vaccinate

eTable 4. Relative Preferences for Vaccination Attributes and Levels, Comparing COVID-19 Beliefs—Regression Results

eTable 5. Relative Preferences for Vaccination Attributes and Levels, Comparing COVID-19 Experience—Regression Results

eTable 6. Latent Class Analysis Showing the Proportion Belonging to Each Class, Along With the Proportion Within Each Class Choosing a Profile Based on the Presence of a Specific Attribute Level

eTable 7. Relative Importance Weights for Choice Attributes (Latent Class Analysis)

eTable 8. Latent Class Analysis by Respondent Characteristics

eTable 9. The Odds of Opting Out by Demographic and COVID-19-Related Experiences, Stratified by Decision-Making for Oneself vs for a Child

eFigure 1. Relative Preferences for Vaccination Attributes and Levels in Limited Population, Comparing Adult and Child Vaccination

eFigure 2. Relative Preferences for Vaccination Attributes and Levels, Comparing Intent to Vaccinate

eFigure 3. Relative Preferences for Vaccination Attributes and Levels, Comparing COVID-19 Beliefs

eFigure 4. Relative Preferences for Vaccination Attributes and Levels, Comparing COVID-19 Experience

eFigure 5. Latent Class Analysis

This supplemental material has been provided by the authors to give readers additional information about their work.

© 2023 Prosser LA et al. *JAMA Netw Open.*

eTable 1. Respondent Characteristics

**Characteristic**	**No. (%)**
Gender	
Male	416 (40.0)
Female	610 (58.7)
Other/prefer not to disclose	14 (1.4)
Age, years	
18-34	300 (28.9)
35-54	361 (34.7)
55-74	324 (31.2)
≥75	55 (5.3)
Race or ethnicity	
Asian	57 (5.5)
Non-Hispanic Black	131 (12.6)
Hispanic	179 (17.2)
Non-Hispanic White	647 (62.2)
Mixed or other	26 (2.5)
Education	
< High school/high school/GED	324 (31.2)
Some college or associate degree	222 (21.4)
Bachelor's degree	337 (32.4)
Advanced degree	157 (15.1)
Household income, $	
Under 34 999	351 (33.8)
35 000 to 74 999	370 (35.6)
≥75 000	319 (30.7)
Self-reported health status	
Excellent	186 (17.9)
Very good	411 (39.5)
Good	290 (27.9)
Fair	124 (11.9)
Poor	29 (2.8)
Respondents thinking of a child of specific age, years	
No	439 (42.2)
0-5	116 (11.2)
6-11	240 (23.1)
12-17	245 (23.6)
Experience with COVID-19	
Yes, confirmed with test	93 (8.9)

© 2023 Prosser LA et al. *JAMA Netw Open.*

Yes, but not confirmed	57 (5.5)
No	852 (81.9)
Don't know/skipped	38 (3.7)
COVID-19 vaccine plans	
I have received or am scheduled to receive the vaccine already	689 (66.3)
I intend to get it as soon as I can	109 (10.5)
I do not intend to get it soon but might sometime in the future	130 (12.5)
I do not intend to ever get the vaccine	112 (10.8)
COVID-19 not as problematic as media presents	
Strongly agree	127 (12.2)
Agree	204 (19.6)
Disagree	304 (29.2)
Strongly disagree	405 (38.9)
Confidence level in answering questions	
Very confident	743 (71.5)
Somewhat confident	276 (26.6)
Not confident	12 (1.2)
They were total guesses	8 (0.8)

© 2023 Prosser LA et al. *JAMA Netw Open.*

eTable 2. Subgroup Analysis

	Adult		Child	
	β	95% CrI	β	95% CrI
Vaccine effectiveness				
60%	0.41	0.00 - 0.80	1.46	1.06 - 1.84
95%	9.59	9.20 - 10.00	8.54	8.16 - 8.94
Mild side effects				
Headache	5.50	5.13 - 5.88	5.37	5.00 - 5.75
Fever, severe chills	4.50	4.12 - 4.87	4.63	4.25 - 5.00
Rare adverse events				
No risk	6.37	5.76 - 6.88	7.01	6.46 - 7.59
Same risk as flu vaccine	4.63	4.06 - 5.27	4.80	4.23 - 5.44
Higher risk than flu vaccine	4.00	2.86 - 5.18	3.18	1.97 - 4.31
Number of doses				
1 dose	5.40	5.03 - 5.77	5.41	5.04 - 5.79
2 doses	4.60	4.23 - 4.97	4.59	4.21 - 4.96
Regulatory approval				
Full approval	6.00	5.61 - 6.37	5.62	5.23 - 6.00
Emergency use authorization	4.00	3.63 - 4.39	4.38	4.00 - 4.77
Waiting time				
1 hour	5.61	4.81 - 6.41	5.51	4.74 - 6.33
2 hours	5.36	5.15 - 6.54	5.37	5.07 - 6.48
4 hours	5.36	4.18 - 5.58	5.36	4.29 - 5.70
8 hours	3.67	1.47 - 5.86	3.76	1.49 - 5.89

Relative preferences for vaccination attributes and levels comparing adult and child vaccination, regression results, excluding respondents who indicated their answers to the questions were “total guesses” (N=8).

Note: Limited population

© 2023 Prosser LA et al. *JAMA Netw Open.*

eTable 3. Relative Preferences for Vaccination Attributes and Levels, Regression Results, Comparing Intent to Vaccinate

	Adult				Child			
	Do not intend to vaccinate		Intend to vaccinate		Do not intend to vaccinate		Intend to vaccinate	
	β	95% CrI	β	95% CrI	β	95% CrI	β	95% CrI
Vaccine effectiveness								
60%	1.70	0.51 - 2.83	0.18	-0.27 - 0.59	2.29	1.03 - 3.53	1.34	0.92 - 1.75
95%	8.30	7.17 - 9.49	9.82	9.41 - 10.27	7.71	6.47 - 8.97	8.66	8.25 - 9.08
Mild side effects								
Headache	5.67	4.60 - 6.82	5.45	5.06 - 5.86	5.64	4.40 - 6.88	5.33	4.94 - 5.73
Fever, severe chills	4.33	3.18 - 5.40	4.55	4.14 - 4.94	4.36	3.12 - 5.60	4.67	4.27 - 5.06
Rare adverse events								
No risk	7.86	6.14 - 9.64	6.23	5.58 - 6.77	8.15	6.29 - 10.01	6.92	6.34 - 7.53
Same risk as flu vaccine	4.98	3.31 - 6.83	4.61	3.99 - 5.29	5.52	3.65 - 7.44	4.74	4.13 - 5.42
Higher risk than flu vaccine	2.16	-1.46 - 5.55	4.16	2.94 - 5.43	1.33	-2.45 - 5.06	3.34	2.05 - 4.53
Number of doses								
1 dose	5.97	4.90 - 7.12	5.38	4.97 - 5.76	6.93	5.64 - 8.18	5.28	4.90 - 5.69
2 doses	4.03	2.88 - 5.10	4.62	4.24 - 5.03	3.07	1.82 - 4.36	4.72	4.31 - 5.10
Regulatory approval								
Full approval	7.52	6.41 - 8.65	5.79	5.40 - 6.22	6.23	4.97 - 7.42	5.56	5.15 - 5.96
Emergency use authorization	2.48	1.35 - 3.59	4.21	3.78 - 4.60	3.77	2.58 - 5.03	4.44	4.04 - 4.85
Waiting time								
1 hour	3.88	1.41 - 6.19	5.85	5.02 - 6.75	3.35	0.74 - 5.85	5.79	4.96 - 6.65
2 hours	6.05	2.40 - 6.41	5.39	5.29 - 6.80	6.16	5.04 - 9.46	5.39	4.86 - 6.35
4 hours	6.09	4.65 - 8.91	5.38	3.89 - 5.39	6.19	2.93 - 7.62	5.38	4.23 - 5.71
8 hours	3.98	-1.52 - 11.55	3.37	1.07 - 5.80	4.30	-2.93 - 11.29	3.44	1.29 - 5.95

© 2023 Prosser LA et al. *JAMA Netw Open.*

eTable 4. Relative Preferences for Vaccination Attributes and Levels, Comparing COVID-19 Beliefs – Regression Results

	Adult				Child			
	COVID-19 is not a problem		COVID-19 is a problem		COVID-19 is not a problem		COVID-19 is a problem	
	β	95% CrI	β	95% CrI	β	95% CrI	β	95% CrI
Vaccine effectiveness								
60%	2.16	1.46 - 2.84	-0.38	-0.89 - 0.11	2.88	2.13 - 3.58	0.89	0.40 - 1.34
95%	7.84	7.16 - 8.54	10.38	9.89 - 10.89	7.12	6.42 - 7.87	9.11	8.66 - 9.60
Mild side effects								
Headache	5.86	5.16 - 6.55	5.41	4.96 - 5.87	5.42	4.68 - 6.15	5.39	4.95 - 5.85
Fever, severe chills	4.14	3.45 - 4.84	4.59	4.13 - 5.04	4.58	3.85 - 5.32	4.61	4.15 - 5.05
Rare adverse events								
No risk	5.86	4.83 - 6.83	6.55	5.88 - 7.24	6.36	5.28 - 7.44	7.19	6.53 - 7.88
Same risk as flu vaccine	3.97	2.87 - 5.06	4.94	4.24 - 5.72	3.81	2.70 - 5.00	5.24	4.56 - 6.02
Higher risk than flu vaccine	5.17	3.11 - 7.30	3.51	2.04 - 4.87	4.83	2.56 - 7.02	2.56	1.10 - 3.91
Number of doses								
1 dose	5.74	5.05 - 6.42	5.34	4.89 - 5.78	6.09	5.36 - 6.82	5.21	4.75 - 5.63
2 doses	4.26	3.58 - 4.95	4.66	4.22 - 5.11	3.91	3.18 - 4.64	4.79	4.37 - 5.25
Regulatory approval								
Full approval	6.13	5.42 - 6.85	6.05	5.59 - 6.52	5.69	4.96 - 6.45	5.72	5.26 - 6.17
Emergency use authorization	3.87	3.15 - 4.58	3.95	3.48 - 4.41	4.31	3.55 - 5.04	4.28	3.83 - 4.74
Waiting time								
1 hour	5.49	4.02 - 6.99	5.71	4.77 - 6.72	3.52	1.97 - 5.11	6.30	5.38 - 7.27
2 hours	5.64	4.52 - 7.00	5.44	5.10 - 6.81	5.68	4.93 - 7.57	5.44	4.85 - 6.55
4 hours	5.65	3.19 - 5.71	5.44	4.06 - 5.79	5.69	4.26 - 6.97	5.43	3.80 - 5.48
8 hours	3.22	0.31 - 8.27	3.41	0.68 - 6.08	5.10	0.36 - 8.83	2.83	0.70 - 5.97

© 2023 Prosser LA et al. *JAMA Netw Open.*

eTable 5. Relative Preferences for Vaccination Attributes and Levels, Comparing COVID-19 Experience – Regression Results

	Adult				Child			
	COVID-19 experience		No COVID-19 experience		COVID-19 experience		No COVID-19 experience	
	β	95% CrI	β	95% CrI	β	95% CrI	β	95% CrI
Vaccine effectiveness								
60%	1.92	1.05 - 2.83	0.01	-0.45 - 0.45	2.96	2.04 - 3.81	1.06	0.61 - 1.49
95%	8.08	7.17 - 8.95	9.99	9.55 - 10.45	7.04	6.19 - 7.96	8.94	8.51 - 9.39
Mild side effects								
Headache	5.24	4.35 - 6.17	5.56	5.15 - 5.99	4.87	3.97 - 5.81	5.48	5.06 - 5.90
Fever, severe chills	4.76	3.83 - 5.65	4.44	4.01 - 4.85	5.13	4.19 - 6.03	4.52	4.10 - 4.94
Rare adverse events								
No risk	5.69	4.32 - 7.03	6.55	5.90 - 7.14	7.51	6.12 - 8.88	6.96	6.32 - 7.57
Same risk as flu vaccine	4.66	3.22 - 6.24	4.63	4.00 - 5.33	3.55	1.97 - 4.97	5.09	4.45 - 5.79
Higher risk than flu vaccine	4.65	1.73 - 7.45	3.82	2.53 - 5.10	3.94	1.15 - 6.91	2.95	1.63 - 4.23
Number of doses								
1 dose	5.40	4.51 - 6.35	5.40	4.98 - 5.80	5.02	4.09 - 5.94	5.49	5.08 - 5.92
2 doses	4.60	3.65 - 5.49	4.60	4.20 - 5.02	4.98	4.06 - 5.91	4.51	4.08 - 4.92
Regulatory approval								
Full approval	6.76	5.83 - 7.72	5.86	5.44 - 6.28	6.46	5.53 - 7.38	5.47	5.04 - 5.89
Emergency use authorization	3.24	2.28 - 4.17	4.14	3.72 - 4.56	3.54	2.62 - 4.47	4.53	4.11 - 4.96
Waiting time								
1 hour	4.41	2.40 - 6.43	5.90	5.04 - 6.81	4.61	2.64 - 6.55	5.81	4.95 - 6.71
2 hours	5.83	4.34 - 7.47	5.41	5.04 - 6.61	5.85	3.11 - 6.35	5.41	5.23 - 6.82
4 hours	5.84	2.88 - 6.14	5.40	4.14 - 5.72	5.86	3.54 - 6.92	5.40	4.09 - 5.67
8 hours	3.93	-0.04 - 10.38	3.29	0.86 - 5.77	3.68	0.18 - 10.71	3.38	0.81 - 5.73

© 2023 Prosser LA et al. *JAMA Netw Open.*

eTable 6. Latent Class Analysis Showing the Proportion Belonging to Each Class, Along With the Proportion Within Each Class Choosing a Profile Based on the Presence of a Specific Attribute Level

	Adults				Children			
	Safety and regulation	Vaccine rejecters	Careful deciders	Convenience	Safety and regulation	Vaccine rejecters	Careful deciders	Convenience
Proportion of population	14%	21%	57%	9%	18%	29%	48%	6%
Vaccine effectiveness								
60%	20%	0%	31%	17%	19%	0%	38%	14%
95%	80%	0%	69%	83%	81%	0%	62%	86%
Opt out	0%	100%	0%	0%	0%	100%	0%	0%
Mild side effects								
Headache	62%	0%	60%	28%	59%	0%	57%	37%
Fever, severe chills	38%	0%	40%	72%	41%	0%	43%	63%
Opt out	0%	100%	0%	0%	0%	100%	0%	0%
Rare adverse events								
No risk	42%	0%	47%	0%	36%	0%	49%	0%
Same risk as flu vaccine	56%	0%	25%	0%	45%	0%	25%	0%
Higher risk than flu vaccine	1%	0%	29%	100%	18%	0%	26%	100%
Opt out	0%	100%	0%	0%	0%	100%	0%	0%
Number of doses								
1 dose	0%	0%	63%	60%	0%	0%	71%	69%
2 doses	100%	0%	37%	40%	100%	0%	29%	31%
Opt out	0%	100%	0%	0%	0%	100%	0%	0%
Regulatory approval								
Full approval	98%	0%	39%	99%	83%	0%	41%	100%
Emergency use authorization	2%	0%	61%	1%	17%	0%	59%	0%
Opt out	0%	100%	0%	0%	0%	100%	0%	0%
Waiting time								
1 hour	0%	0%	12%	82%	0%	0%	14%	100%
2 hours	58%	0%	24%	0%	51%	0%	21%	0%
4 hours	10%	0%	32%	0%	9%	0%	34%	0%
8 hours	33%	0%	33%	18%	40%	0%	31%	0%
Opt out	0%	100%	0%	0%	0%	100%	0%	0%

© 2023 Prosser LA et al. *JAMA Netw Open.*

eTable 7. Relative Importance Weights for Choice Attributes (Latent Class Analysis)

	Adults				Children			
	Safety and regulation	Vaccine rejecters	Careful deciders	Convenience	Safety and regulation	Vaccine rejecters	Careful deciders	Convenience
95% VE	0.8034	0.0002	0.6910	0.8302	0.8068	0.0001	0.6173	0.8640
1 day of headache, fatigue	0.6188	0.0002	0.6001	0.2761	0.5858	0.0001	0.5748	0.3696
No rare adverse events	0.4247	0.0001	0.4667	0.0005	0.3643	0.0001	0.4916	0.0006
1 dose	0.0011	0.0002	0.6324	0.6020	0.0008	0.0001	0.7053	0.6909
1 hour wait	0.0001	0.0001	0.1154	0.8225	0.0001	0	0.1449	0.9975
Full FDA approval	0.9812	0.0002	0.3943	0.9899	0.8266	0.0001	0.4088	0.9967

© 2023 Prosser LA et al. *JAMA Netw Open.*

eTable 8. Latent Class Analysis by Respondent Characteristics

	Adults					Children					All respondents
	Safety and regulation	Vaccine rejecters	Careful deciders	Convenience	P-value	Safety and regulation	Vaccine rejecters	Careful deciders	Convenience	P-value	
Age					0.687					0.414	
18-34	15.9	18.6	55.9	9.5		18.6	25.8	49.5	6.06		28.9
35-54	14.0	22.5	53.6	10.0		18.4	28.0	48.0	5.49		34.7
55-74	15.8	22.0	52.0	10.3		16.7	31.8	44.5	7.04		31.2
75+	15.8	18.2	57.6	8.5		19.4	30.9	43.0	6.67		5.3
Gender					0.172					0.846	
Male	16.0	20.0	54.4	9.6		18.2	26.8	48.9	6.15		40.0
Female	14.6	22.0	53.4	9.9		17.9	30.0	45.9	6.22		58.7
Other	16.7	4.8	64.3	14.3		16.7	28.6	47.6	7.14		1.4
Race/Ethnicity					0.645					0.285	
White	14.9	21.7	53.2	10.2		17.5	30.0	46.3	6.18		62.2
Hispanic	16.9	16.9	56.0	10.3		20.2	22.7	49.4	7.68		17.2
Black	14.3	23.3	55.2	7.3		16.5	31.8	47.3	4.39		12.6
Asian	16.4	18.7	55.0	9.9		20.5	24.0	49.7	5.85		5.5
Other	14.1	25.6	50.0	10.3		16.7	33.3	43.6	6.41		2.5
Education					<.0001					<.0001	
High school	14.0	26.4	50.7	8.9		15.6	36.7	42.7	5.02		31.2
Associates degree	15.5	26.7	49.4	8.4		16.7	36.0	41.0	6.31		21.4
Bachelor’s degree	15.7	15.4	58.6	10.4		20.3	21.0	52.2	6.59		32.4
Advanced degree	16.4	13.6	57.2	12.8		20.0	18.7	53.7	7.64		15.1
Income					<.0001					<.0001	
Under $34,999	13.9	27.8	50.3	8.1		16.2	35.9	42.5	5.38		33.8
$35,000 to $74,999	15.8	21.4	52.1	10.8		16.9	28.5	47.5	7.1		35.6
$75,000 or more	16.1	13.1	60.1	10.8		21.1	21.1	51.7	6.06		30.7
Health status					0.050					0.123	
Excellent	14.6	19.6	57.1	8.7		15.9	26.0	52.6	5.59		17.9
Very good	15.6	18.7	56.4	9.4		19.3	28.1	47.0	5.54		39.5
Good	15.4	22.3	50.4	11.9		18.5	28.4	45.6	7.52		27.9
Fair/Poor	14.7	26.4	50.2	8.7		16.0	34.4	43.3	6.22		14.7
Thinking of child of specific age					0.015					<.0001	
No	14.1	24.8	51.6	9.5		16.0	34.9	42.9	6.17		42.1
Yes											
Age 0-5 years	18.0	20.6	54.2	7.3		17.7	36.5	42.0	3.77		19.3
Age 6-11 years	15.6	16.9	56.9	10.6		20.3	22.5	50.8	6.39		40.0
Age 12-17 years	15.5	18.4	55.1	11.0		19.3	20.3	53.2	7.21		40.7%
COVID-19 experience					0.008					0.003	
Yes	19.2	15.0	55.3	10.5		22.2	20.6	50.8	6.49		15.0
No	14.5	22.0	53.7	9.7		17.3	30.1	46.5	6.15		85.0

© 2023 Prosser LA et al. *JAMA Netw Open.*

eTable 8 cont. Latent Class Analysis by Respondent Characteristics

	Adults					Children					All respondents
	Safety and regulation	Vaccine rejecters	Careful deciders	Convenience	P-value	Safety and regulation	Vaccine rejecters	Careful deciders	Convenience	P-value	
COVID-19 vaccination intentions					<.0001					<.0001	
Already vaccinated/intend to vaccinate	17.14	8.63	62.36	11.87		20.71	16.37	55.39	7.53		76.8
Delay vaccination/no intention to vaccinate	8.89	61.67	26.25	3.19		9.03	69.44	19.72	1.81		23.3
COVID-19 not as problematic as media presents					<.0001					<.0001	
Agree/strongly agree	13.01	34.55	45.43	7.01		13.31	44.11	38.31	4.27		31.8
Disagree/strongly disagree	16.25	14.64	57.93	11.18		20.17	21.54	51.18	7.1		68.1

© 2023 Prosser LA et al. *JAMA Netw Open.*

eTable 9. The Odds of Opting Out by Demographic and COVID-19-Related Experiences, Stratified by Decision-Making for Oneself vs for a Child

	Adult vaccination OR (95% CrI)	Adults for childhood vaccination OR (95% CrI)	P-value of interaction term (difference in strength of association between adult and child vaccination decision-making)
Age			0.268
18-34 vs 35-54	0.57 (0.40, 0.82)	0.59 (0.40, 0.86)	
55-74 vs 35-54	0.87 (0.64, 1.20)	1.03 (0.74, 1.45)	
75+ vs 35-54	0.76 (0.44, 1.29)	1.03 (0.56, 1.88)	
Gender			0.111
Male vs Female	0.86 (0.66, 1.13)	0.73 (0.55, 0.98)	
Other vs Female	0.31 (0.09, 1.09)	0.86 (0.21, 3.44)	
Race/Ethnicity			0.808
Hispanic vs Non-Hispanic White	0.84 (0.58, 1.22)	0.74 (0.50, 1.11)	
Non-Hispanic Black vs Non-Hispanic White	1.27 (0.85, 1.92)	1.35 (0.89, 2.05)	
Non-Hispanic Asian vs Non-Hispanic White	1.10 (0.58, 2.08)	1.06 (0.56, 2.01)	
Other vs Non-Hispanic White	1.15 (0.51, 2.56)	1.23 (0.55, 2.74)	
Education			0.661
High School vs Bachelors	1.63 (1.16, 2.28)	1.87 (1.30, 2.70)	
Associates vs Bachelors	1.67 (1.17, 2.38)	1.90 (1.30, 2.78)	
Advanced vs Bachelors	0.95 (0.62, 1.47)	0.99 (0.62, 1.56)	
Income			0.165
<$35,000 vs >$75,000	2.02 (1.40, 2.92)	1.57 (1.05, 2.33)	
$35,000 - $75,000 vs >$75,000	1.45 (1.03, 2.05)	1.26 (0.88, 1.82)	
Parenthood Status			0.0002
Parent of Child 0-5 years vs Not Parent	0.74 (0.49, 1.13)	1.04 (0.67, 1.63)	
Parent of Child 6-11 years vs Not Parent	0.60 (0.42, 0.85)	0.56 (0.39, 0.80)	
Parent of Child 12-17 years vs Not Parent	0.66 (0.47, 0.92)	0.46 (0.32, 0.66)	
COVID-19 Experience			0.294
Yes vs No	0.58 (0.39, 0.85)	0.49 (0.32, 0.76)	
COVID-19 Beliefs			0.896
COVID is a problem vs COVID is not a problem	0.27 (0.21, 0.36)	0.28 (0.20, 0.37)	

© 2023 Prosser LA et al. *JAMA Netw Open.*

eFigure 1. Relative Preferences for Vaccination Attributes and Levels in Limited Population, Comparing Adult and Child Vaccination

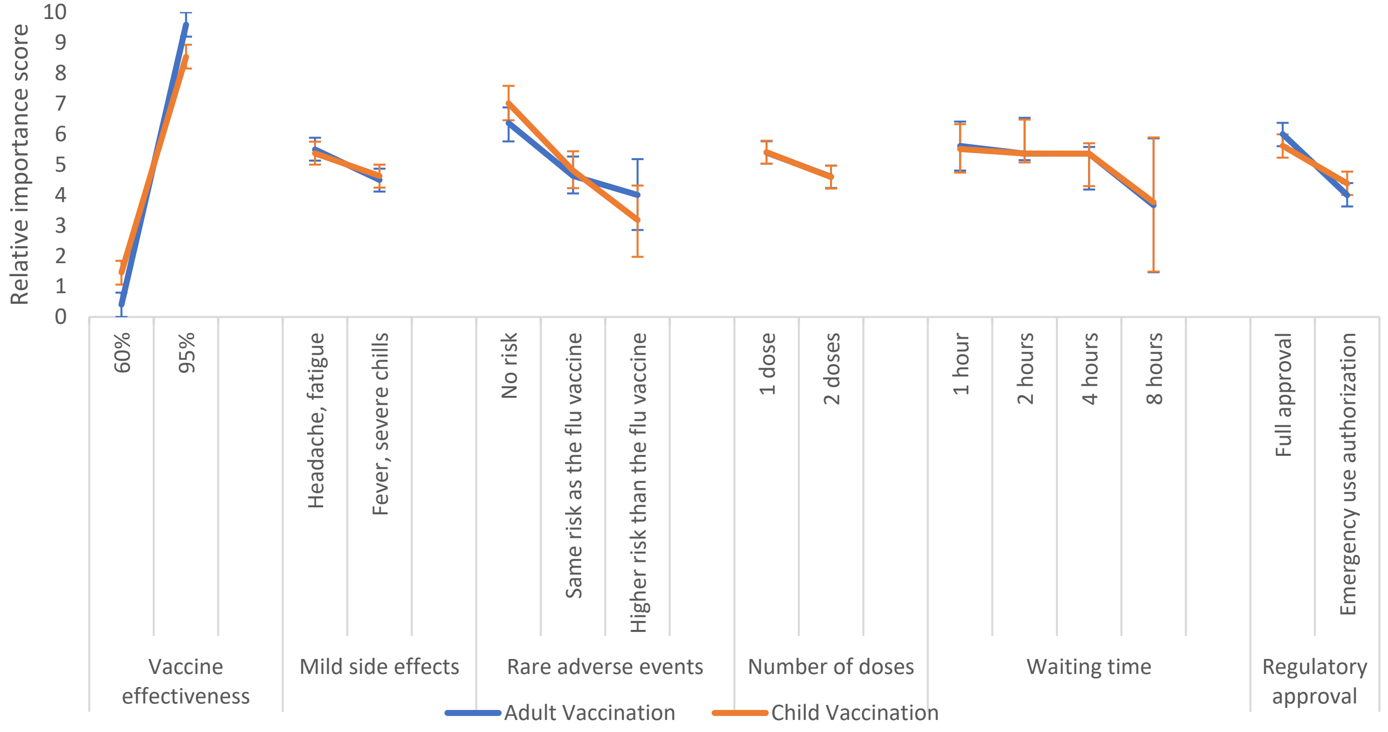

Note: Limited population excludes respondents that indicated their answers to the questions were “total guesses” (N=8).

© 2023 Prosser LA et al. *JAMA Netw Open.*

eFigure 2. Relative Preferences for Vaccination Attributes and Levels, Comparing Intent to Vaccinate

a. Adult

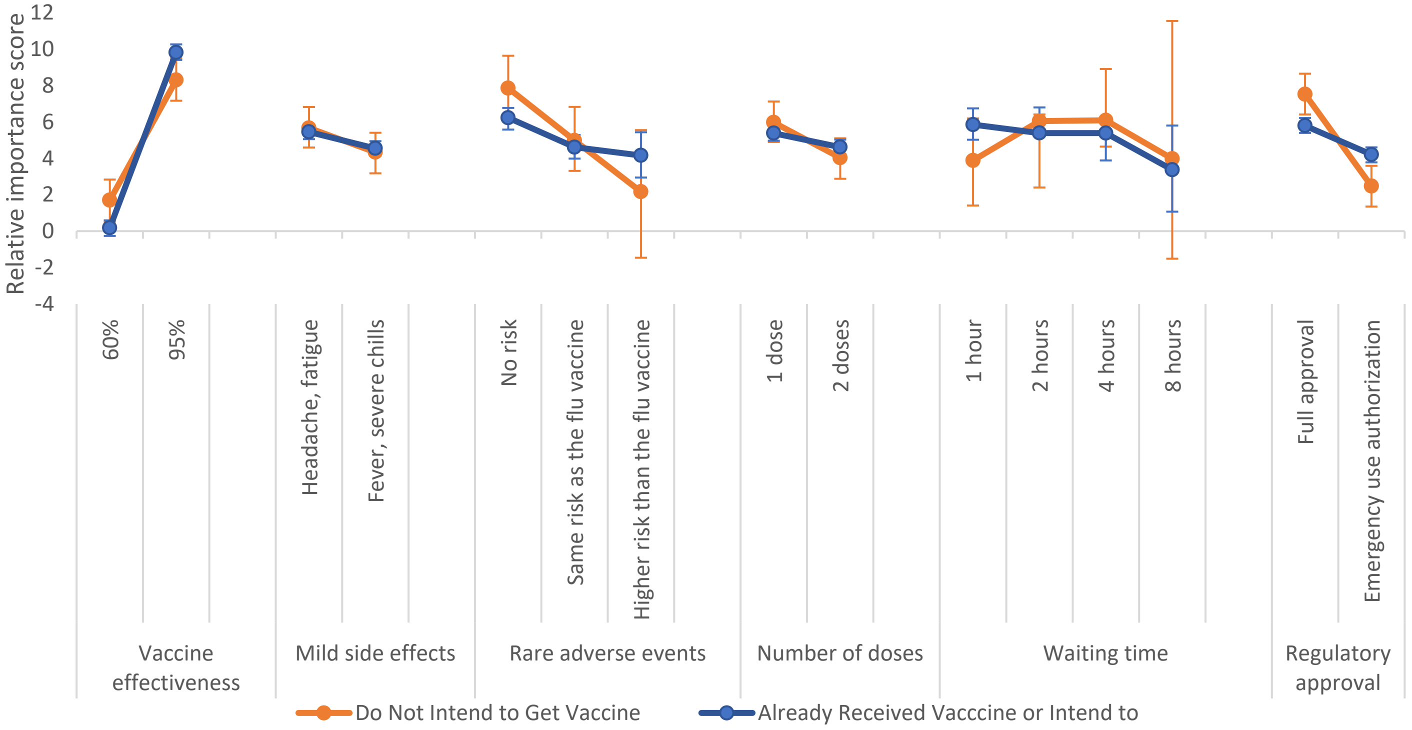

© 2023 Prosser LA et al. *JAMA Netw Open.*

b. Child

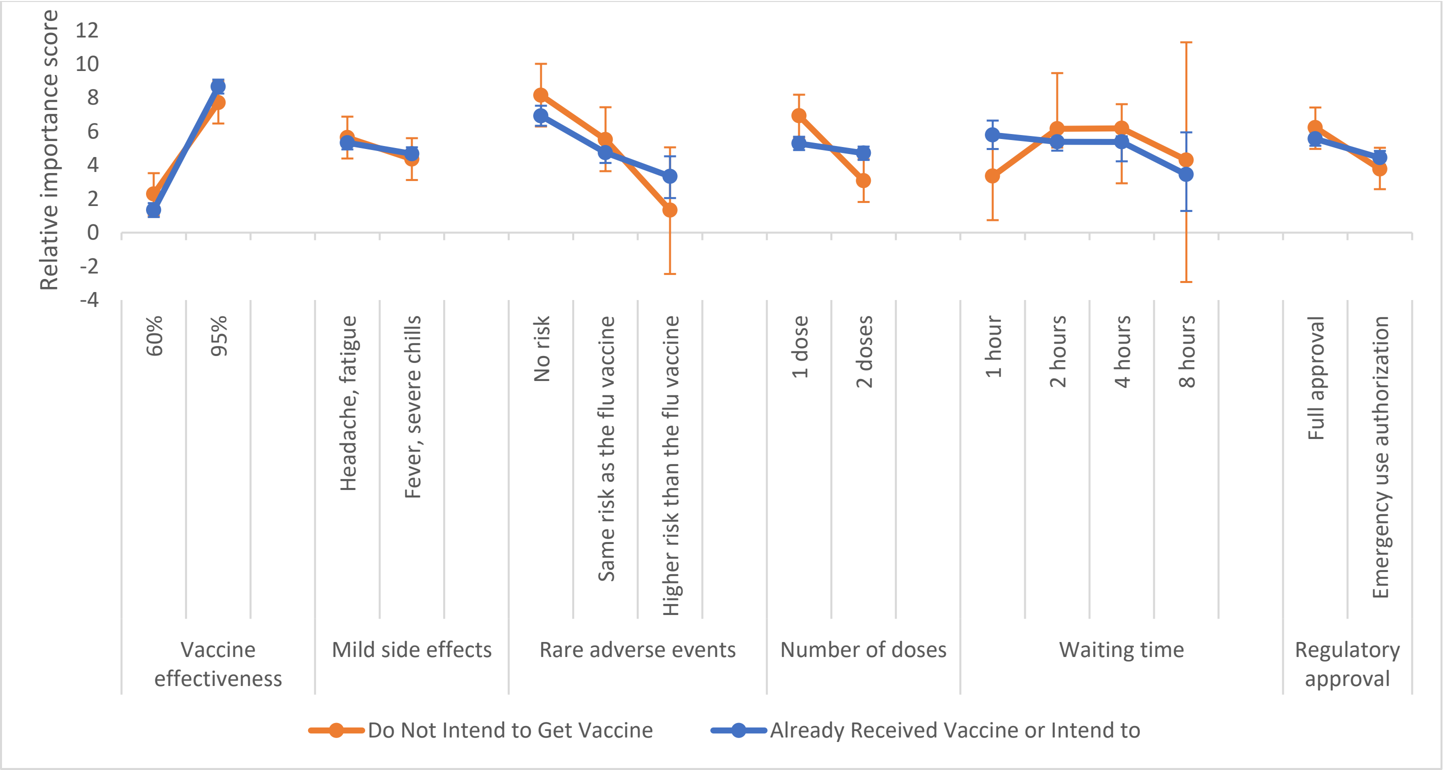

© 2023 Prosser LA et al. *JAMA Netw Open.*

eFigure 3. Relative Preferences for Vaccination Attributes and Levels, Comparing COVID-19 Beliefs

a. Adult

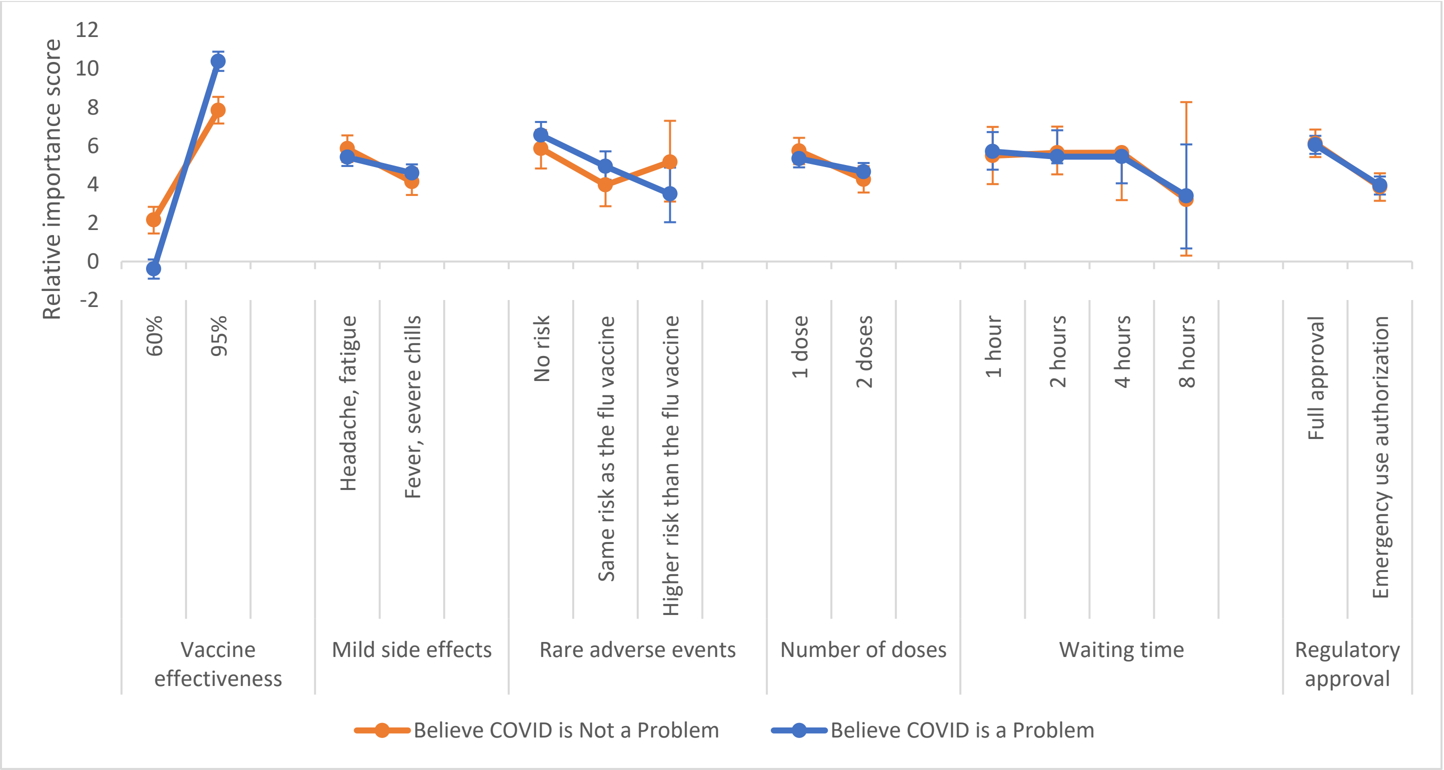

© 2023 Prosser LA et al. *JAMA Netw Open.*

b. Child

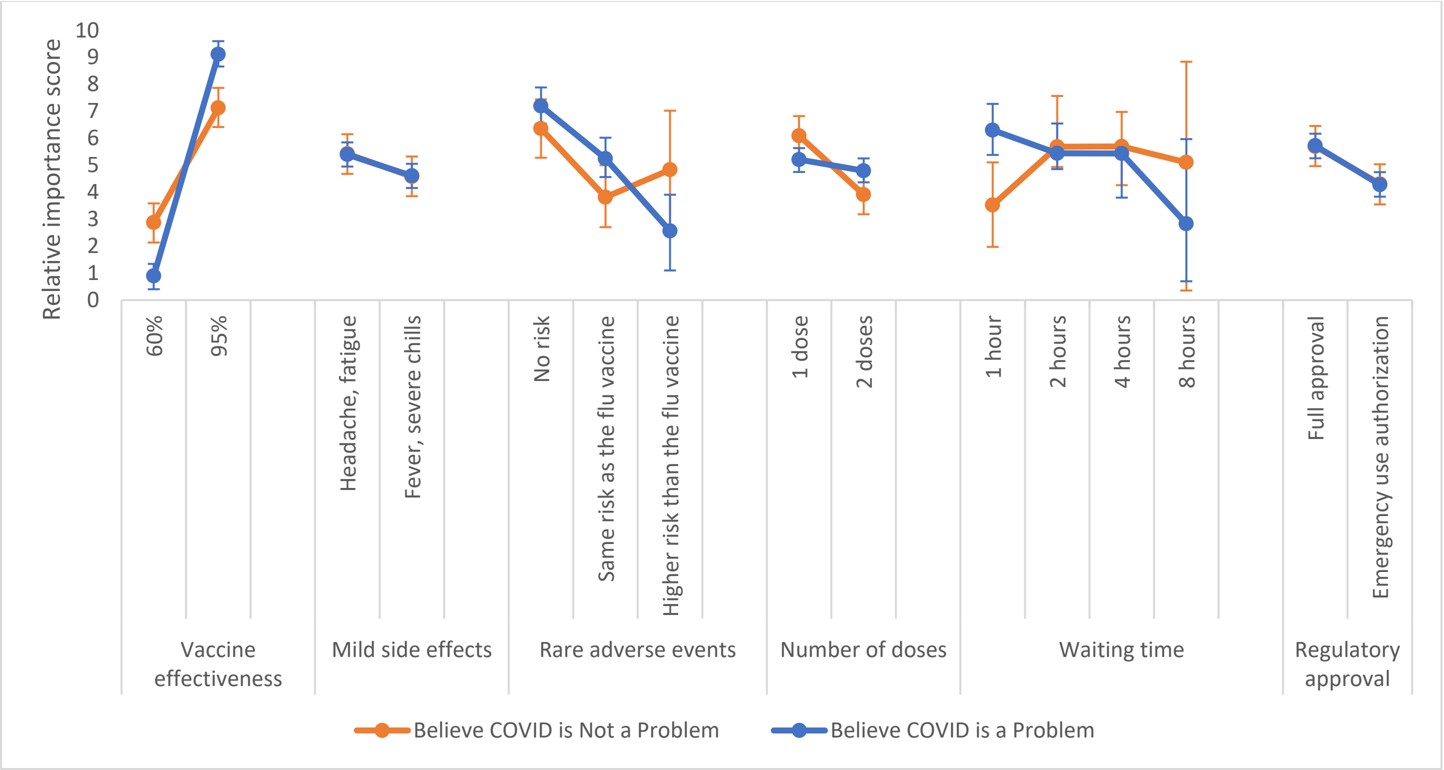

© 2023 Prosser LA et al. *JAMA Netw Open.*

eFigure 4. Relative Preferences for Vaccination Attributes and Levels, Comparing COVID-19 Experience

a. Adult

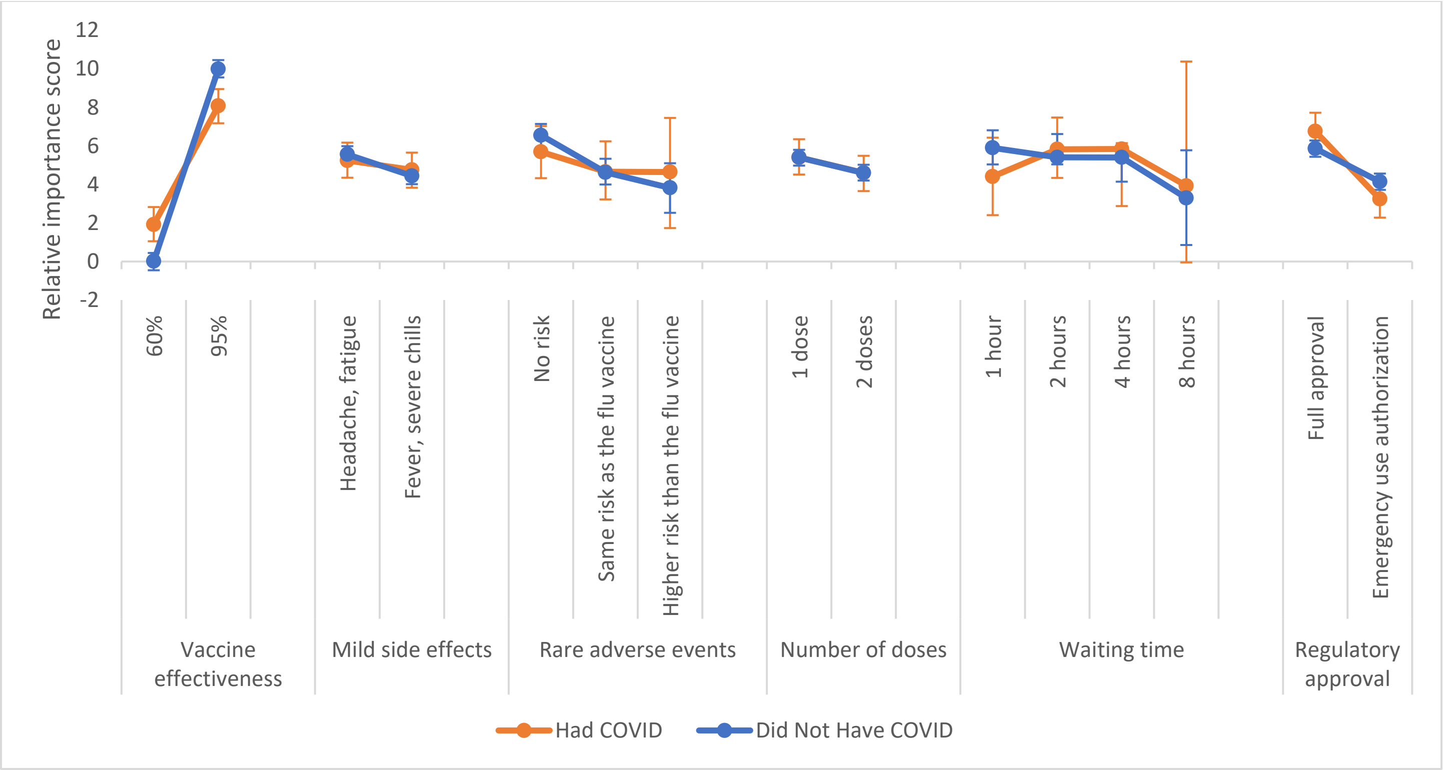

© 2023 Prosser LA et al. *JAMA Netw Open.*

b. Child

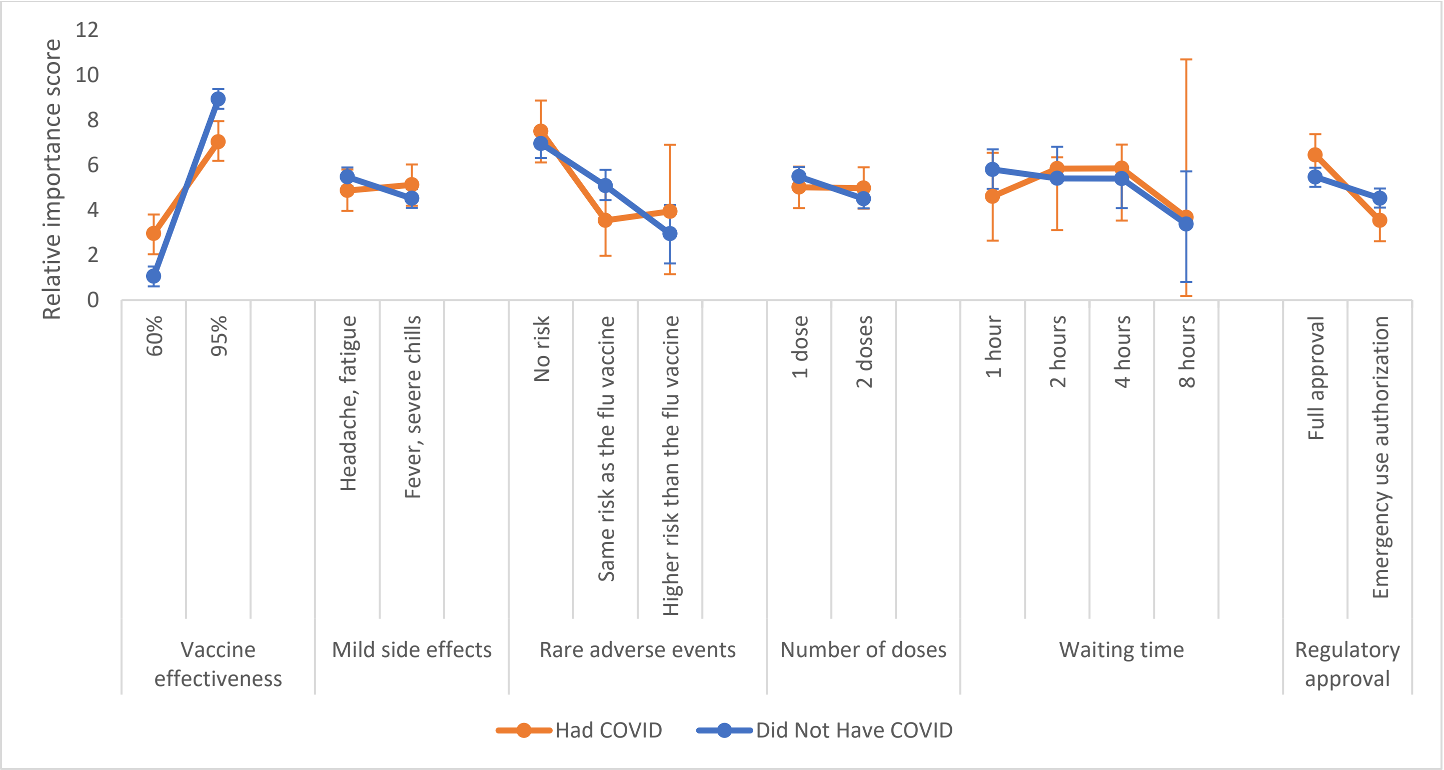

© 2023 Prosser LA et al. *JAMA Netw Open.*

eFigure 5. Latent Class Analysis

a. Adult

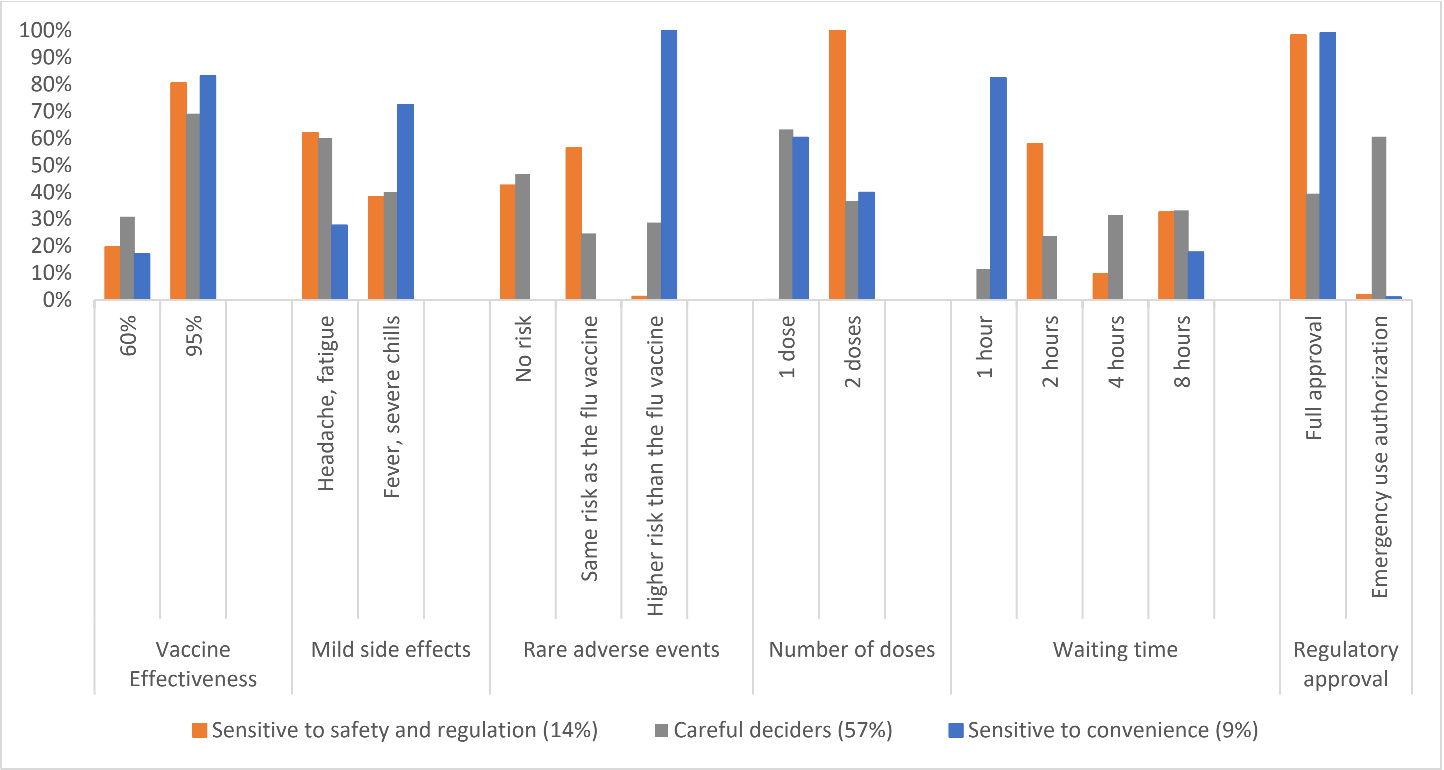

Note: Vaccine rejecters represented 21% of the population. Percentages do not add to 100 due to rounding.

© 2023 Prosser LA et al. *JAMA Netw Open.*

b. Child

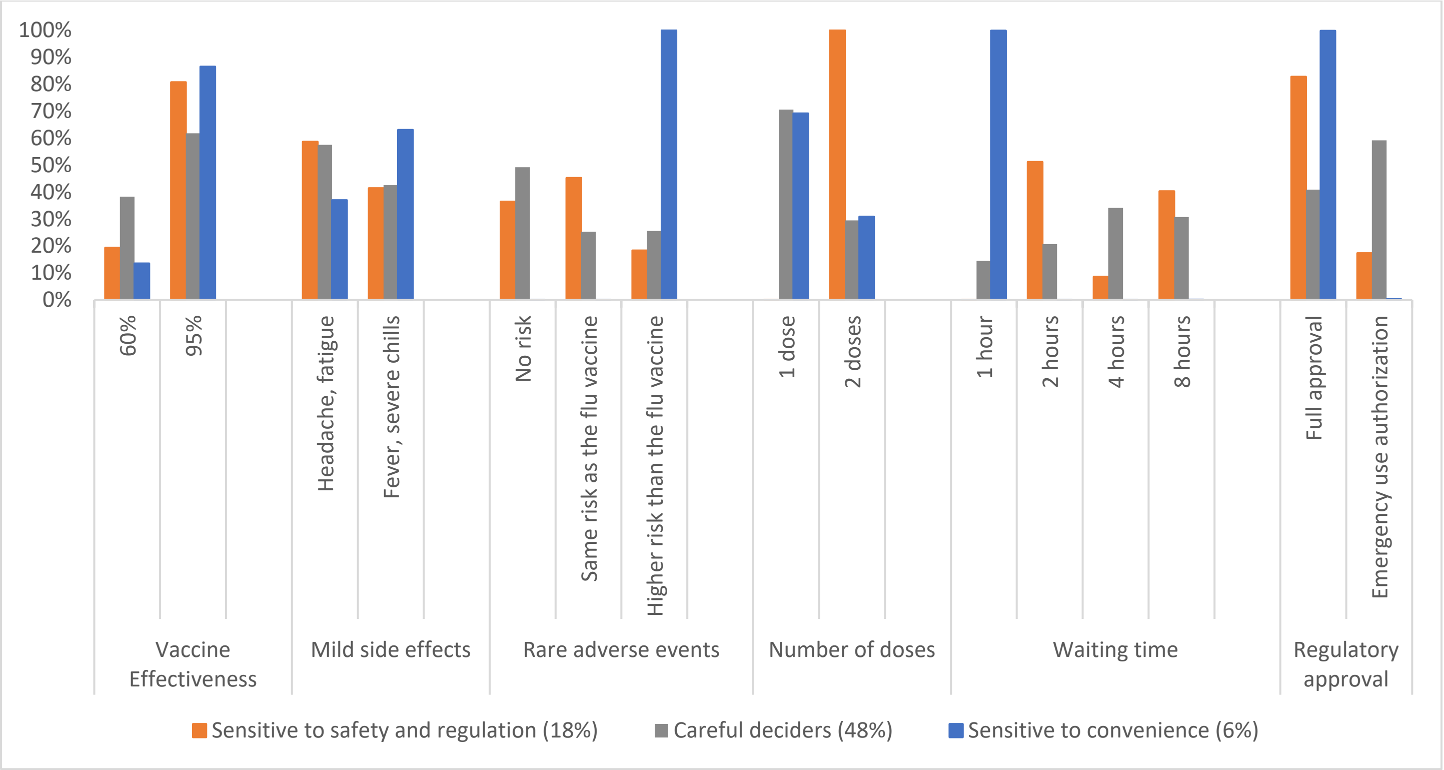

Note: Vaccine rejecters represented 29% of the population. Percentages do not add to 100 due to rounding.

© 2023 Prosser LA et al. *JAMA Netw Open.*
