# Supplementary material for: A Discrete Choice Analysis Comparing COVID-19 Vaccination Decisions for Children and Adults
Source: JAMA Netw Open. 2023 Jan 30;6(1):e2253582. doi: 10.1001/jamanetworkopen.2022.53582 (PMC9887501; doi:10.1001/jamanetworkopen.2022.53582)
Supplement: Supplement 2. — Data Sharing Statement [file jamanetwopen-e2253582-s002.pdf]

Data Sharing Statement

Prosser. A Discrete Choice Analysis Comparing COVID-19 Vaccination Decisions for Children and Adults. *JAMA Netw Open*. Published January 30, 2023.
doi:10.1001/jamanetworkopen.2022.53582

Data
Data available: Yes
Data types: Deidentified participant data, Data dictionary
How to access data: Data are available by contacting the authors at lisapros@umich.edu
When available: With publication

Supporting Documents
Document types: None

Additional Information
Who can access the data: Researchers whose proposed use of the data has been approved.
Types of analyses: For academic purposes.
Mechanisms of data availability: With signed data access agreement
Any additional restrictions: None
